# Amalgam Filling Technic*This is an extract from a valuable paper by Dr. Harper, which is published in full in the December *Journal N. D. A*.

**Published:** 1920-01

**Authors:** Wm. E. Harper


### AMALGAM FILLING TECHNIC *

BY WM. E. HARPER, D.D.S.

The heretofore insurmountable and legitimate objection to its proper and more extended use can be removed by a proper amalgam technic, with the possibility of assured stability. The great tooth-preserving attribute of amalgam, its strength and resistence to attrition, low cost, and comparative ease of manipulation, whatever the condition or wherever the position of the cavity, demands a square deal.

In my contributions on this subject made during the past five years, I have formulated and insisted upon the following steps, in the order presented, as a standardized amalgam technic, essential to adaption, strength, and stability:

* This is an extract from a valuable paper by Dr. Harper, which is published in full in the December *Journal N. D. A.*

PROPORTIONS

Need not be exact but mercury should be plentiful, sufficient to avoid any trace of dryness in the mixing and just within the other extreme of liquid plasticity.

Either extreme is unfavorable to thorough amalgamation.

When the proportions have been decided upon by a trial mix, it will save time and material to use the small amalgam balance sold for that purpose.

The amount of mercury used in making a mix has nothing whatever to do with the excess mercury in the finished filling.

MIXING

All high grade alloys amalgamate rather slowly. It requires thorough rubbing of the particles in the presence of mercury to develop thorough amalgamation, conditions that prohibit a hand mix. Mixing is best accomplished by rapid rubbing in a deep mortar for a minimum time of three minutes. A measure of time is the only dependable guide to a thorough mix.

Thorough mixing is necessary to attain that plasticity most favorable to adaptation. It is also necessary to the greatest strength of the filling, and it is the means by which we may practically eliminate that movement immediately following the placing of the filling. This early movement is common to all alloys.

Mercury may be added or expressed during the mixing to meet the requirements stated.

PLASTICITY

In all average large and proximo-occlusal cavities the amalgam mass must be extremely plastic during the filling of the first two-thirds of the cavity to insure adaptation in the use of high grade alloys.

This important requirement is best accomplished by retaining sufficient of the excess mercury (commonly removed during the final mixing or kneading) to be removed after it has served its purpose, by thorough, forceful, and orderly packing.

The excess mercury generally removed during the final mixing is not dangerous to the stability of the filling, because this mercury is a surplus always removed by very ordinary packing force.

The excess mercury that requires our special attention is the final excess; the complete removal of this dangerous excess is absolutely and solely dependent upon thorough, forceful, and orderly condensation.

Decided plasticity and the importance of uniform condensation are two operative details, not previously recognized by the profession as essential to results, that must be accepted if we would make, with reasonable uniformity, nonleaking and stable amalgam fillings.

### TAMPING

Was designed and has proved to be an aid to uniform condensation. Tamping is done by lightly tapping each piece of amalgam added with the plugger until it spreads as a uniformly thick, dense, and plastic layer in contact with the surrounding walls, and well into the angles, then we may proceed to condense. This procedure provides uniform bulk and plasticity under each thrust of the plugger. Experience shows a material improvement in adaptation when this detail is carried out.

### PACKING

The packing should be as forcible and thorough as possible and applied with the greatest possible uniformity. The most practical means of accomplishing this in the mouth is by an orderly stepping of the plugger.

Following the detail of tamping, we start the packing of each piece of amalgam with a medium or large plugger at the center of the filling, stepping a little less than its diameter in one direction in a gradually enlarging circle until the cavity walls are reached. The final condensation of each piece of amalgam is done by stepping the cavity walls.

To step the cavity walls we use the smallest plugger (size about twelve by eighteen-tenths of a millimeter in diameter) stepping a little less than its long diameter immediately against the walls and well into the angles once around the cavity.

With an orderly packing, the excess mercury is always driven in one direction, outward, where it is at all times free to come to the surface to be removed as it appears.

Packing without order results in irregular condensation. Those parts of the filling thoroughly condensed will contain no excess mercury, and, as a result will be abundantly strong; the parts of the filling less thoroughly condensed will contain excess mercury, and, as a result, the filling will be weak and unstable with corresponding possible defects in adaptation.

With an orderly packing the final excess mercury finds its last lodgment immediately against the cavity walls and particularly in the angles; at which points it will exert its worst effects if allowed to remain. Forcibly stepping the cavity walls is the one procedure that will most certainly remove this dangerous excess and at the same time perfect the adaptation.

In irregular cavities orderly stepping must be carried out as far as it is practicable.

The operating details of condensation are the most exacting and difficult to acquire because they must be carried out simultaneously. To develop the skill necessary to uniform results, demands our most thoughtful consideration until correct habits have been formed.

As the filling approaches completion, the amalgam should be gradually less plastic, until at the finish the excess mercury may be pinched out forcibly to the point where it still remains a workable mass.

The cavity should be filled with an excess, covering well all margins, after which the surface should be thoroughly condensed with the largest plugger. This final condensation may be done with pluggers sufficiently large to cover the whole filling, in combination with the heaviest possible mallet force; or the entire filling should be forcibly compressed with the thumb, holding the amalgam against all margins at the same time for a few moments. This will express the final excess mercury.

The filling should now remain undisturbed for about three minutes, after which it should be trimmed and carved to correct proximal and occlusal form, as far as this is possible; and the surface may be made smooth by light burnishing, and the use of soft brush wheels and pumice. To be given the final polish at a subsequent sitting.

### THE MATRIX

The heavy packing force required to insure the complete removal of excess mercury makes the use of the matrix imperative in all cavities in which one or more of the surrounding walls are missing.

The matrix may be held by ligature, or by use of one of the mechanical retainers sold for that purpose. It should be held securely and should be reasonably well adapted to the tooth at all points along the cavity margins.

It is very much easier to avoid a large bulk of amalgam in the interproximate space and overlap at the gingival margin than it is to trim it off during or after the setting of the amalgam.

For this purpose, I insert a round tapering wood toothpick from the lingual to wedge the matrix solidly against the gingival margin. This wedge will assist materially in preserving the interproximate space and will save much time in trimming the filling to form.

The matrix must be so held that it may be adjusted, or stretched, to restore the contact point when building the filling; and, wherever necessary and possible, sufficient separation should be made to secure enough room in the interproximate space to restore its function.

The importance of restoring the interproximate space, contacts, tooth form, and the polishing of an amalgam filling, and the technic involved in this phase of amalgam work, requires much skill, and is of such importance and extent as to justify separate consideration.

The amalgam technic I have presented during the past five years will be found to meet all of the requirements necessary to practically uniform results in adaptation, strength and stability; and, insofar as I am capable of the task, the procedure has been reduced to the simplest form consistent with results, and will not add more than three to five minutes time taken by the average careful amalgam operator.

For the benefit of those who may not find the time or inclination to read the full reports of the investigations, I beg to correct some of the old-time theories that have been disproved.

Exact laboratory tests have definitely shown that alloys do not deteriorate with age either before amalgamation or after completion of the filling.

Plentiful use of mercury during the mix is necessary to maximum strength and stability, and does not affect the amount remaining in the finished filling.

The more thorough the mix in the mortar up to the time of six minutes, the stronger and the more stable the filling; but the increase in strength after three minutes is so small that it does not justify the extra time.

The more mercury actually rubbed into the alloy the stronger the filling. The great wrong is due to our failure to remove the mercury not amalgamated.

Bulk changes, developed by time and apparent to the naked eye, made from representative high-grade alloys, are entirely due to excess mercury in the finished filling.

Practically every well-known high-grade alloy contains zinc, irrespective of which thousands of permanently good amalgam fillings have been made. This fact clearly proves that zinc alone is not responsible for the movement.

That important attribute, adaptability to cavity walls, is lost when crepitus develops in amalgam, because amalgam in that condition breaks as it is compressed, and these minute breaks can not be eliminated by subsequent packing. Crepitating amalgam packs like snow, bridging the defects as snow bridges the air spaces contained, which can not be eliminated until subjected to very heavy pressure. The more sloppy the snow the more dense and hard the snowball can be made.

If while making the filling the amalgam mass becomes stiff, more mercury may be added and kneaded into the mass to secure the desired plasticity without impairing the strength of the filling.

The excess mercury retained after making the mix to insure a decided plasticity, varies with the size and form of the cavity, or the time required to fill it. This excess, commonly removed during the final kneading, is in no wise dangerous, because it is always the first expressed during the packing.

The dangerous excess is the final excess that will remain in the mass after forcible squeezing during the final kneading, which will always become apparent under thorough and forcible condensation.

Smooth plasticity is not a guide to a thorough mix, smooth plasticity develops long before we reach a condition of thorough amalgamation. Vigorous mixing in the mortar for a minimum time of three minutes is the only dependable guide to a perfect mix.

It would not seem out of place to ask another question: Why do dentists so commonly neglect the essential details of cavity preparation, restoration of proximal and occlusal form and contact, and perfection of finish in their amalgam work?

I would answer this question by saying that the public have been educated to believe that amalgam is undependable because of the common discoloration. This lack of confidence has caused many to base their charge on the low cost of the material instead of the professional service. And insufficient remuneration has reduced operative skill to a very low level.

If this explanation is correct, it is a deplorable situation, and I trust that the presentation of a practical and successful amalgam procedure will awaken our consciences to the injustice to both operator and patient, and will lead to the adoption of a charge for real professional service, which will give to this most valuable filling material a square deal.

